# Energy modeling to compensate for the seasonal lack of electrical and thermal energy depending on the different climates of Iran

**DOI:** 10.1016/j.heliyon.2023.e20455

**Published:** 2023-09-26

**Authors:** Saeed Ahmadi-Kaliji, Ahmad Hajinezhad, Alireza Kashani Lotfabadi, Reza Fattahi, Seyed Farhan Moosavian

**Affiliations:** a Department of Renewable Energies and Environment, Faculty of New Sciences and Technologies, University of Tehran, Tehran, Iran

**Keywords:** Energy policy modeling, Climatic and environmental analysis, District heating adaptability, Combined heat and power plants, Heat pumps

## Abstract

Renewable energy sources are in focus for environment-friendly power generation when compared to non-renewable sources. Modeling an energy system of a statistical population can shed light on the possibilities and potential of using renewable resources. In this study, energy modeling of 4 provinces of Iran with different climates is done for 2020 and 2032. The lack of energy caused by seasonal climatic impacts is compensated for by using renewable energy systems. The modeling of three different scenarios is considered to indicate different policies in each energy system strategy. The energy system's past data is gathered and analyzed to predict future data, and then the 2032 energy system is modeled using EnergyPLAN. The results show that there will be a shortage of electrical energy in summers in hot & humid and hot & dry climates, while the energy shortage for cold and temperate & humid climates is the heating demand in winters. Three scenarios of business as usual (BAU), using maximum possible renewable energy (S1), and changing the structure of the energy supply system (S2) are considered with their specification. The results indicate that by using S1, 61.42 TWh of primary energy sources (PES), and by using S2, 136.7 TWh of PES consumption is reduced. Also, for the same scenarios, 29.98 Mt less CO_2_ is emitted for all climates. The climatic analysis illustrates that using solar in hot & humid and hot & dry, wind and geothermal in cold, and hydropower in hot & humid and temperate & humid climates produce the most amount of renewable potential which not only compensates the lack of seasonal energy but also replace 8% of the total energy needed, previously supplied by fossil fuels. Totally for the 4 provinces, 3250 MW of hydropower, 5625 MW of solar, 650 MW of wind, and 100 MW of geothermal energy are considered while other provinces with the same climate could benefit too based on their geographical specification.

## 1 Introduction

Global warming and climate change are among the most important challenges of humankind in the present century [[1], [2], [3]]. Greenhouse gases (GHG) are the main reason for these challenges which are the product of burning fossil fuels. Power generation produces approximately half of the total emissions [[4], [5], [6], [7]]. Renewable resources have considerably less GHG emissions than non-renewable ones. Thus, renewable power generation has been greatly considered recently. Utilizing cheap and sustainable energy resources could cause economic growth due to their renewability and high performance [[8], [9], [10]]. Recently, policymakers have paid lots of attention to renewable resources in Iran. However, the renewable supply of the current energy system of Iran is far from balancing the total energy demand. Oil price fluctuation adds another reason to consider moving through 100% renewable energy consumption. The reason is to lower GHG emissions and prevent a 1.5 °C rise in global temperature [[11], [12], [13]]. Recent policies indicate that analyzing local energy systems is important because of the climatic considerations of each province. Also, the analysis of the energy system should contain all demanding sectors including residential, industry, transportation, etc on a local basis. This consideration leads to a better understanding and policy-making of each section based on the current and future technologies [[14], [15], [16]]. In this study, energy system modeling of different climates in Iran is under investigation on a local basis by maximizing the importance of renewable energy resources and waste energy. The effects of climatic conditions, the energy balancing policy, and the renewable resource assessment on the energy system will be discussed to illustrate and guide future energy policies. The study's objective is to indicate three scenarios of future energy systems which will be compared together. These scenarios could define future energy policies and their impacts.

Modeling the energy system of each statistical population should be revised gradually due to the quick changes in technology and policies. So, the subject literature contains recent developments in energy systems modeling and also future energy system prediction. Goh et al. [17] used a data analysis method for wind energy to predict future power generation. They used the ELM model for low-frequency and CNN for high-frequency data. Arevalo et al. [18] used EnergyPLAN to model the demand distribution of the Galapagos island for 2030 and 2050. They figured the island's energy could be supplied by wind and photovoltaic (PV) energy by 2050. Kumari et al. [19] used FLCNN neural network method to predict energy supply methods using a hybrid solar and wind energy system. They also used the JAYA algorithm for the optimization of the system.

Energy resource assessment is another field of major recent studies done on different parts of the world to assess each form of renewable energy locally. Abdul et al. [20] compared wind, solar, hydropower, and biomass energy supply methods for a society. They suggested that hydropower efficiency is the highest with the wind being second best. Qiu et al. [21] studied the renewable resources of Tibet in two scenarios. They implemented geographic constraints which caused a reduction of 1.31 GWh of renewable energy potential. They suggested using hydropower based on the physiological parameters of the location. McKenna et al. [22] assessed the renewable energy potential of England considering the latest energy policies and suggested that 1324 TWh of onshore wind, 153 TWh of roof PV, and 1200–7093 TWh of ground PV systems can be utilized.

Some studies also investigated the future of renewable energy systems based on several environmental and economic policies. Some studies used small-scale renewable energy production like municipal solid waste (MSW) power generation [23] and biohydrogen energy production [24]. Ahmad et al. [25] reviewed the determinants of renewable energy sources in Pakistan. Sanchez et al. [26] suggested the integration of wind and solar energy in the future and discussed the problems of utilizing the system. They advised the integration of periodic and non-periodic renewable energy supply methods with both economic and sociological storage possibilities in Spain, where they suggested the system based on seasonal performance. Chen et al. [27] studied an integrated energy supply system containing PV, ground source heat pump (GSHP), and electrical heater for a district heating demand. Their results indicated a 10% improvement in energy efficiency while CO_2_ emissions decreased by approximately 2%. However, their economic analysis illustrated a $0.03/KWh rise in the net cost of energy. Dong et al. [28] studied different renewable scenarios using GAN and machine learning methods. Their simulations resulted in a controlled method that can use their scenarios to model the energy system. Geleta et al. [29] used the GWO method to minimize the total wind and solar energy supply methods. They found the optimized number of solar collectors, wind turbines, and batteries to support their fixed demand.

Some studies used decision-making systems to find renewable energy resources and the effects of utilizing them on the local energy system of Iran. Razeghi et al. [30] used a multi-criteria decision-making process to find the desalination potential of Iran utilized with solar power by reverse osmosis. The city of Khansar was chosen to have the best possible conditions for this purpose. In another study, Razeghi et al. [31] used multi-criteria decision-making to find the best places for a wind farm desalination system. They needed 0.38 KWh for the desalination process which was supplied from the wind farm of 550 KW wind turbines. They figured that 63% of the country's water needs could be supplied. Mirhosseini et al. [32] assessed the wind potential of Semnan province. They used different levels of evaluation and figured that 5 cities of Biarjmand, Damghan, Garmsar, Semnan, and Shahroud are capable of wind power generation. They concluded that 2000 MW of wind power capacity could be utilized. Alamdari et al. [33] reviewed the studies done on the measurements of wind speed at different elevations. They figured that 68 potential sites are suitable for wind power generation. Najafi et al. [34] discussed the solar energy outlook of Iran. They stated that the average irradiation of 2200 KWh/m^2^ could be integrated with the solar panels to generate 9 TWh of electricity. They predicted the potential of renewable energy in Iran for 2030 to be 2.8 GWh. Noorollahi et al. [35] used a multi-criteria decision-making system to locate the potential of wind energy in Iran. They figured that 28% of the proposed area is suitable for power generation. Peyvandi et al. [36] investigated the intensity of GHG emissions for electricity production in Iran. They stated that renewable power generation has 2.2 Ton/KWh CO_2_ emissions while fossil fuel power plants emit 506 Ton/KWh CO_2_. They also figured that solar is the best possible replacement for fossil fuels.

Energy security is another topic of recent studies. Razeghi et al. [37] integrated renewable resources with CHP power plants to improve energy security. The CHP plants use mostly biomass, natural gas, and coal. They figured that by using district heating, 31.4% less fuel would be needed. Noorollahi et al. [38] reviewed the two decades of geothermal power generation in Iran. Rajaei [39] forecasted the future climate in the Tajan watershed. She suggested a 1.6–2.2 °C rise in the watershed temperature until 2040. Several other studies were done on the renewable wind, solar, geothermal, hydropower, and biomass potential of Iran [[40], [41], [42]].

This study uses EnergyPLAN [43,44] to model the energy system of four different provinces of Iran with different climatic circumstances. Four provinces of Mazandaran (temperate & humid), Ardabil (cold), Yazd (hot & dry), and Hormozgan (hot & humid) are considered to show climatic differences in their energy systems. A data analysis method is implemented to analyze previous data and predict future years. Based on the difference in these climates, certain energy shortages will occur at different times of the period studied. The goal of the paper is to design and model a sustainable energy system using renewable resources to cover the mentioned energy shortage and also the inevitable rise of energy consumption. Three different scenarios are chosen based on three different policies to indicate the best possible scenario considering all situations. The novelty of the study is considering the climatic effects of the regions studied on the seasonal shortage of energy while compensating for this shortage with renewable resource supply systems based on each region's climate and also waste heat recovery. Also, for the first time, the impacts of three different policies on the energy system of each province are discussed and the best scenario in each case is suggested.

## 2 Modeling and raw data

**Fig. 1:**
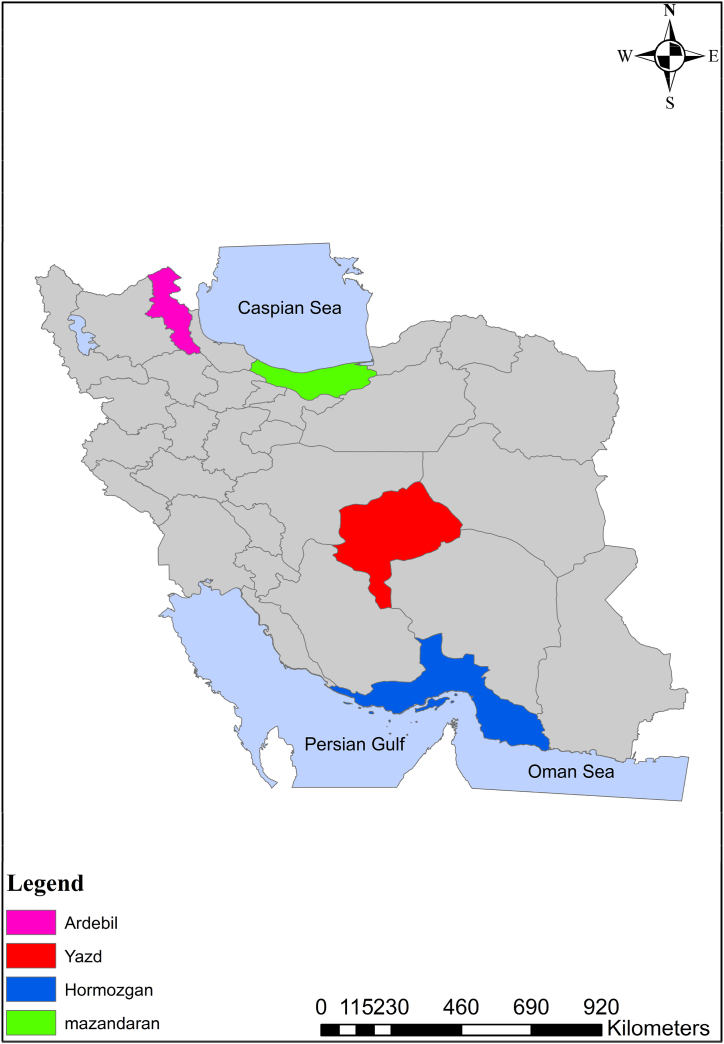
Location of the provinces studied [48].

In the present study, four different climates of Iran are modeled in terms of energy demand and supply methods. Fig. 1 shows the map of Iran where the provinces stated are colored. Mazandaran province is at the top of the map south of the Caspian Sea. The area of 23,756 km^2^ contains 3.3 million population based on recent statistics. Ardabil is located in the northwestern part of Iran with an area of 17,953 km^2^ which is a residence for 1.3 million people. Yazd is in the middle part of Iran, where 1.1 million people live in an area of 76,469 km^2^. Hormozgan is in the southern part of the country (North of the Persian Gulf and Oman Sea) with a 1.8 million population and a 70,697 km^2^ area [[45], [46], [47]]. Table 1 contains the provinces’ climatic details (see Table 2).

**Table 1:** Climatic properties of each province [48].

Province	Climate	Summer Design Temperature (°C)	Summer Relative Humidity (%)	Winter Design Temperature (°C)	Winter Relative Humidity (%)
Mazandaran	Temperate & Humid	33.6	63	−3	92
Ardabil	Cold	29.7	52	−22	78
Yazd	Hot & Dry	40	24	−9.7	71
Hormozgan	Hot & Humid	40.5	57.5	7.5	80

**Table 2:** Current situation of power plants of Mazandaran and Yazd [48].

	Mazandaran				Yazd			
	Capacity (MW)		Electricity Production (MWh)		Capacity (MW)		Electricity Production (MWh)	
	Fossil Fuel	Hydropower	Fossil Fuel	Hydropower	Fossil Fuel	Solar PV	Fossil Fuel	Solar PV
2012	2262	14.6	13312.7	16.9	1152.8	0	4402.7	0
2013	2270	14.6	12,000	34.7	1519.9	0	5257.5	0
2014	2270.4	534.6	11491.1	41.1	1669.3	0	6210.2	0
2015	2294.9	1054.6	12,370	533	1997.9	0	8875.8	0
2016	2304.9	1054.6	12035.7	543.1	2330.1	0	10166.9	0
2017	2314.5	1054.6	11310.3	756.7	2534	4	11363.2	0
2018	2410.9	1054.6	11647.9	912.6	2718	24	11704.5	3.7
2019	2784.9	1054.6	12117.7	892.2	2743.1	62.5	13691.6	66.8
2020	2826.8	1057.2	11579.1	1006	2762.8	62.5	13209.6	115.2

As shown in Table 1, Mazandaran has a temperate & humid climate. This specific climatic condition is due to the adjacent Caspian Sea and heavy rains during the year. Ardabil has 130 days of below-freezing temperatures. So, the heating energy demand per population is higher than in other provinces. Yazd contains mostly the middle deserts of Iran, making the daily temperature difference greater than in other provinces. Hormozgan consumes more electricity than other provinces due to the cooling demand in summer.

**Fig. 2:**
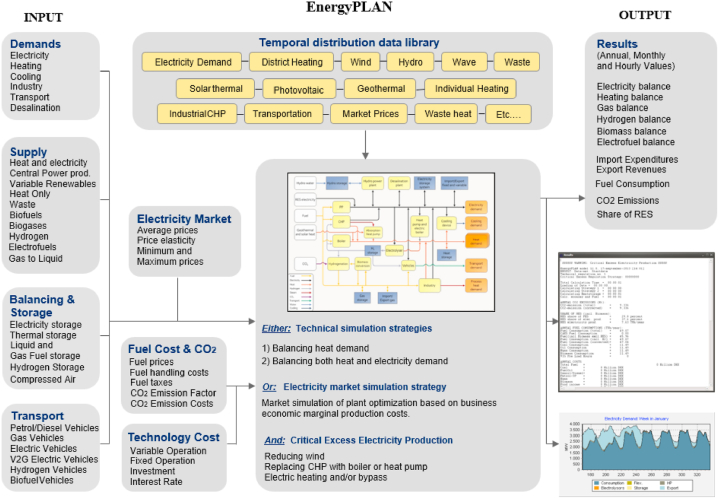
Schematic of EnergyPLAN model used in the current study.

As shown in Fig. 2, EnergyPLAN is an advanced energy system analysis computer model that uses a bottom-up technique [44]. It uses statistical population inputs of residential, industrial, transport, power generation demand, and supply data. Also, it uses heating, cooling, and electricity consumption and distribution. Then, it calculates the given data by the modeling techniques and indicates the energy system characteristics including primary energy consumption and CO_2_ emissions. The society in question could be local, national, regional, or worldwide [43].

**Fig. 3:**
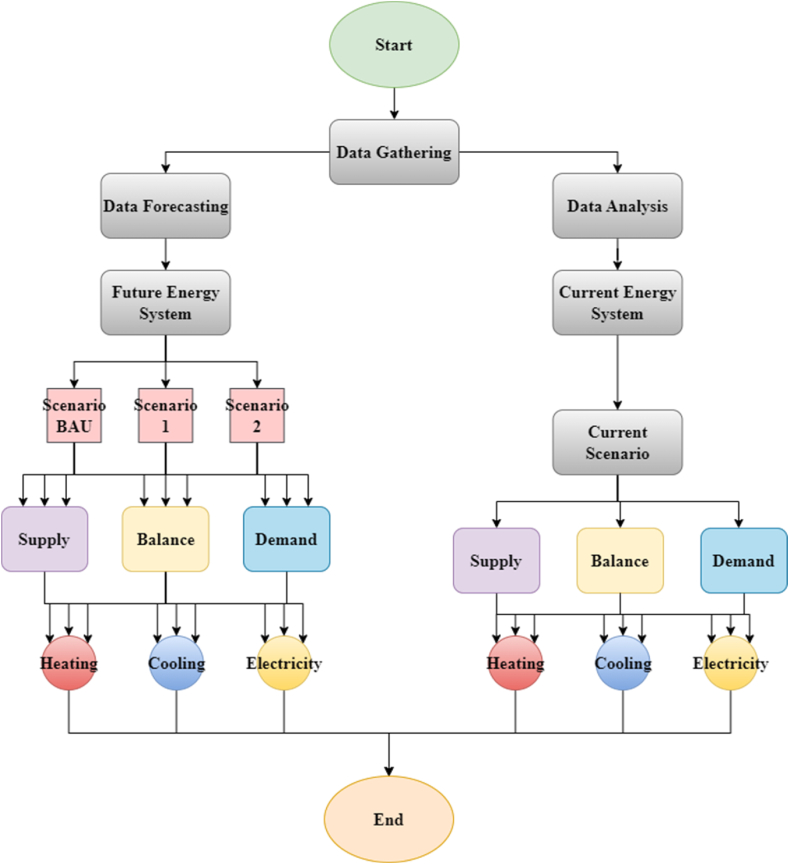
Process of the study [48].

Fig. 3 shows the process used in this study to model the energy system of the provinces. First, the energy system data of the last eight years is assembled, and the current energy system is modeled. The past and present data are then used to predict future energy system data and then the future energy system is modeled under 3 different assumptions. A linear regression method (extrapolation) is used for the prediction by finding a 9-year rate of increase or decrease. Based on the data gathered, the first scenario (BAU) is assumed to be the exact policies chosen before which is called business as usual. All of the energy shortage is supplied by renewable sources in the second scenario (S1). The third scenario (S2) is considered to be the energy system with the least CO_2_ emissions. All assumptions for all scenarios are based on national standards. Also, improving the fuel quality and using electric vehicles (EVs) are considered for S2.

**Fig. 4:**
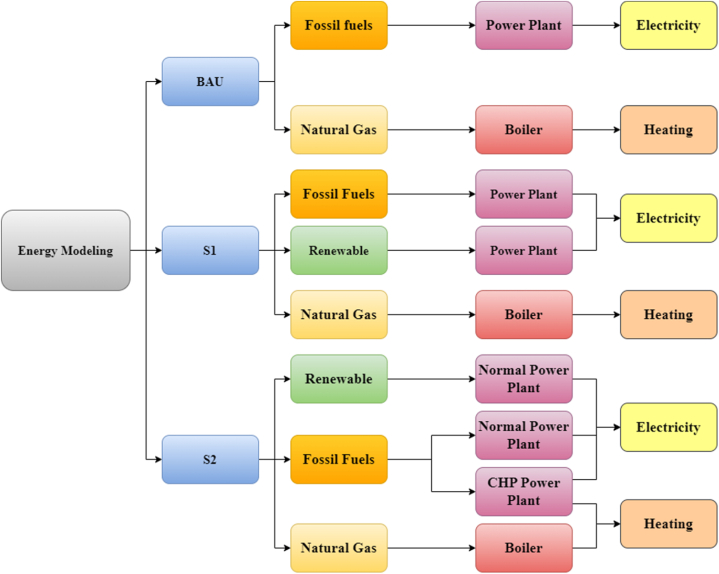
Description of scenarios.

Fig. 4 describes the scenarios mentioned. As shown, the increasing demand for energy in the future is compensated for by renewable energy sources in S1. Also, S2 suggests using CHP power plants as much as possible to maximize energy efficiency.

The mathematical extrapolation is done as follows:

(1) $$\Delta_{i}=\left|x_{i}-x_{i+1}\right|$$

(2) $$\bar{x}=\frac{\sum_{i=1}^{n-1}\Delta_{i}}{n}$$

(3) $$x_{n+1}=x_{n}\mp \bar{x}$$

First, the deviation of each 2 years' data is calculated. Then, the mean deviation is calculated. Finally, the following year's data is calculated by adding the last data to the mean deviation.

**Fig. 5:**
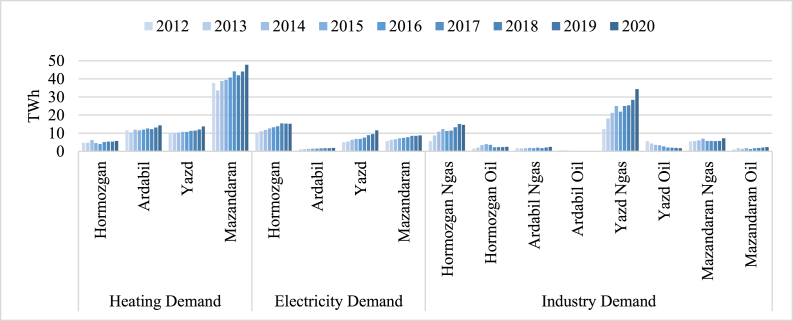
Electricity, heat, and industry demand of previous years [48].

As shown in Fig. 5, the raw data of previous years' heating, electricity, and industry demands are gathered. Mazandaran has the highest heating demand while Yazd consumes the most amount of natural gas for industrial purposes. This means Yazd could be considered an industrial province. So, the energy system policies should implement electrification more. Also, the electrification process should be utilized with renewable resources to supply the industry's demand as well. As mentioned, the Mazandaran climate is temperate & humid accompanied by cold winters. The main reason for this high heating demand is the high population per area. Hormozgan consumes the most electricity in all provinces. The reason could be the cooling demand in the summer. The total electricity, heating, and industry demands are increasing in every province.

Technologies, like heat pumps, could be used for electrification purposes for heating since the temperature needed is below the boiling point of water, and heat pumps are perfectly compatible with multiple renewable sources [49]. The regular fuel for heating and power plants is natural gas in Iran. So, the demand for natural gas is continuously increasing. Also, the dropping demand for oil in the industry is because of the higher power plant capacity, which consumes both oil products and natural gas as burning fuels.

**Fig. 6:**
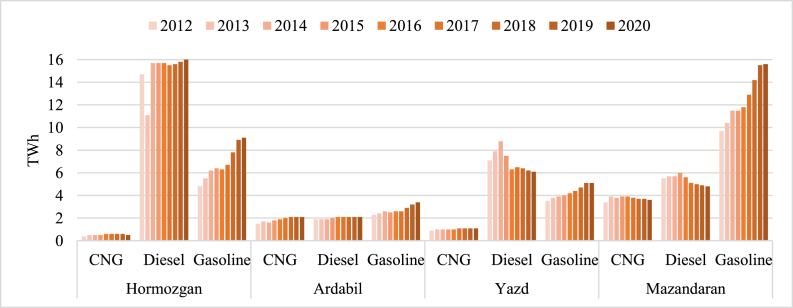
Transport demand of previous years [48].

Fig. 6 illustrates the different fuels used for transport usage in 4 provinces in the previous years. Again Mazandaran consumes the most amount of gasoline due to the population, while Hormozgan consumes the most amount of diesel which could be due to the several import-export ports which contain lots of trucks and other heavy vehicles. CNG produces less amount of CO_2_ because of the lower carbon content in the composition than diesel and gasoline. However, current policies indicate using gasoline and diesel-burning engines in the province the most. Using electric vehicles (EVs) or improving fuel quality could lower the amount of GHG emissions in this sector.

Table 3 indicates the current power plants of 4 provinces. The capacity and yearly power generation of each province's power plants are written. As shown, renewable energy contributes less energy to the energy system than non-renewable energy. Only the hydropower plant in Mazandaran generates power considerably, which is an awful situation since the potential of renewable resources in Iran is much higher than in countries with much bigger renewable production. A country wind atlas project estimates that according to available wind power generation technology, economic conditions, and wind potential, the power generation potential in Iran is about 15,000 MW [50]. Different potential geothermal regions of north and northwest Iran contain 91.08 × 1018 J of thermal energy [51,52]. Iran's total potential for producing solar energy is estimated to be around 40,000 GWh. This concludes that the provinces could benefit from renewable resources a lot more than they are by using the correct policies.

**Table 3:** Current situation of power plants of Ardabil and Hormozgan [48].

	Ardabil						Hormozgan			
	Capacity (MW)			Electricity Production (MWh)			Capacity (MW)		Electricity Production (MWh)	
	Fossil Fuel	Hydropower	Wind	Fossil Fuel	Hydropower	Wind	Fossil Fuel	Solar PV	Fossil Fuel	Solar PV
2012	1002.3	13.1	0	1774.2	67.2	0	2569.9	0	12173.1	0
2013	1002.3	13.1	0.6	2002.5	55.7	0.8	2569.9	0	12481.1	0
2014	1002.3	13.1	0.6	2488.5	0	0.6	2595.1	0	12184.3	0
2015	1002.3	13.1	1.3	2447	0	0.4	3267.5	0	13550.9	0
2016	1002.3	13.1	1.3	2309.6	26.5	0.7	3267.4	0	15045.3	0
2017	1002.3	13.1	1.3	2577.9	42.4	0.8	3267.4	0	15335.5	0
2018	1002.4	13.1	1.3	3047.9	52.2	0.6	3229.9	38.4	15902.2	0.6
2019	1008.4	13.1	1.3	3007.3	57.9	0	3497.4	10	13691.3	16.7
2020	1008.4	13.1	1.3	2895.1	59.5	0	3804.4	10	13815.1	16.9

### 2.1 Energy policy modeling

The scenarios chosen are carefully assumed based on the energy policies indicated in several international conferences. BAU is the same policy that is now undertaken in Iran. Using 71% natural gas and 29% oil products in power plants makes them the lead primary energy used in Iran. Three types of power plants are being used. Gas-cycle, steam-cycle, and combined heat and power (CHP) are all used in the provinces. However, the CHPs turn the waste heat to power in a different steam-cycle phase of the power plants. The residential and industrial heating is supported by boilers, and the transportation is based on euro-4 standards. The BAU scenario indicates the future of the provinces using extra power plants for the growing power demand and also using more heating boilers for residential and industrial heating. S1 uses the amount of renewable resources needed to compensate for the energy shortage in each region. However, the heating and transportation systems are the same as BAU. This indicates that the policies governing the maximum renewable energy production could have the results which will be discussed in part 3. In S2, all present power plants are changed to CHP, while the waste heat will be directly used for residential heating by utilizing district heating tools. Also, to have a wide possibility of changing the supply system for balancing the heat and electricity demand in seasonal performance for different climates, an amount of heat pump systems are considered. Of course, the electricity and the excess heat are manageable, though the capacities of CHP and renewable plants are implemented in such a way as to lower the CO_2_ emissions to the least possible number. These policies are later discussed and elaborated on more.

## 3 Results

First, the future energy system is calculated based on data extrapolation. Then, the power plants are implemented according to the scenarios chosen for 2032 and the results indicate the best policies accordingly.

### 3.1 Energy system modeling

The demands of each scenario are calculated based on the climates of the provinces considered.

**Fig. 7:**
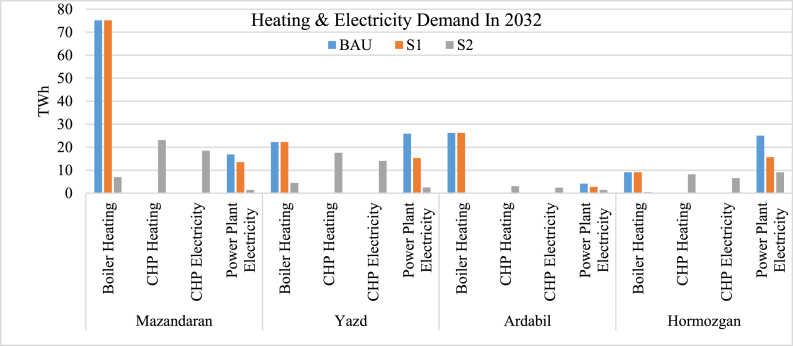
Heating and electricity demand for future.

Fig. 7 demonstrates the electricity production of non-renewable power plants and also different heating supply systems. As shown in the BAU scenario, the electricity demand is supplied by the regular gas-cycle or steam-cycle power plants, while in S1, renewable power plants are added. In S2, most non-renewable power plants changed into combined heat and power plants (CHPs). When using CHP plants, waste heat is used for power generation. Instead, it is utilized by district heating to supply a part of the heating demand.

**Fig. 8:**
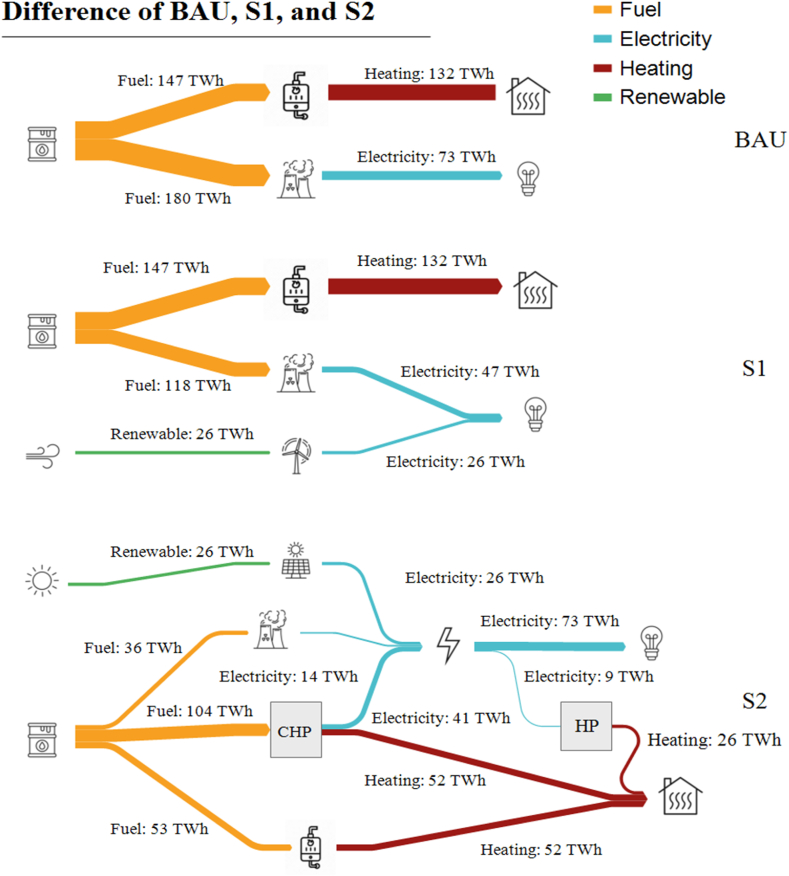
Changes between energy supply in different scenarios.

As shown in Fig. 8, in S2, the mentioned district heating system is utilized accordingly, which resulted in a 70 TWh decrease in boiler heating for Mazandaran. The same criteria are 20, 25, and 10 TWh for Yazd, Ardabil, and Hormozgan, respectively. This means by using a district heating system supported by waste CHP heat, most of the residential heating demand could be supplied. The CHP plants also use non-renewable fuels. However, balancing the fuel consumption for such power plants leads to lower total non-renewable fuels used both for electricity generation and heating supply. In this scenario, the capacity of CHP plants is lower than the previous power plants’ capacity since the waste heat is not turned into electricity with a certain efficiency. So, renewable sources should be utilized to supply the remaining electricity needed.

**Fig. 9:**
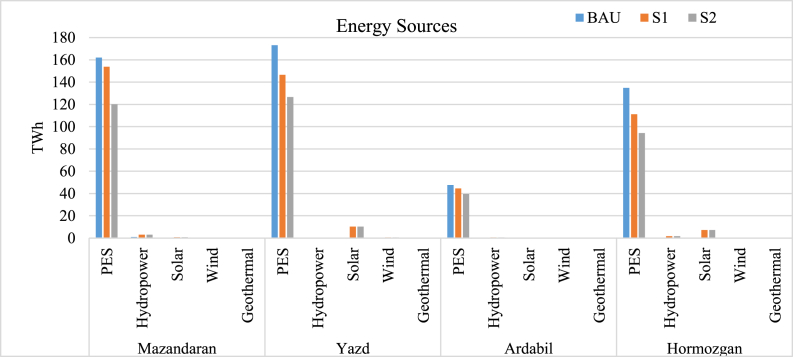
Primary and renewable energy source.

Fig. 9 describes primary and renewable energy sources for yearly power generation for each province. As shown, S2 has the least primary energy consumption, whereas renewable sources provide more than BAU. However, renewable energy generation still contributes to a low percentage of the total supplied energy which is the product of renewable energy resource assessment. Yazd and Hormozgan's solar power generation is higher due to their adaptable climate, while Mazandaran hydropower is more than other provinces. Ardabil uses geothermal energy more than other renewable sources, more than other provinces since the geothermal resource assessment demonstrates a better performance of such energy systems in the northwestern part of Iran.

The results indicate that by using S1, 61.42 TWh of primary energy sources (PES), and by using S2, 136.7 TWh of PES consumption is reduced in all climates combined. This is due to the utilization of waste energy in CHP plants and the additional renewable energy sources used. This method could be applied to all of the current power plants to prevent extra ones to be built.

### 3.2 Renewable impacts

As mentioned, the scenarios are designed to compensate enough energy for the current and future lack of energy in each climate. So, renewable resources are also designed for this purpose. The capacities of each renewable source used, are compared to the maximum capacity of renewable resources in each climate, which could suggest that the design is feasible. Mazandaran's new renewable power plants are a 2000 MW hydropower, a 250 MW solar, and a 100 MW wind power plant. Yazd's renewable plants are chosen as a 3065 MW solar and a 250 MW wind. The renewable plants for Ardabil are a 100 MW geothermal, a 250 MW hydropower, and a 100 MW wind, while in Hormozgan, a 2310 MW solar, a 1000 MW hydropower, and a 200 MW wind power plant are considered.

**Fig. 10:**
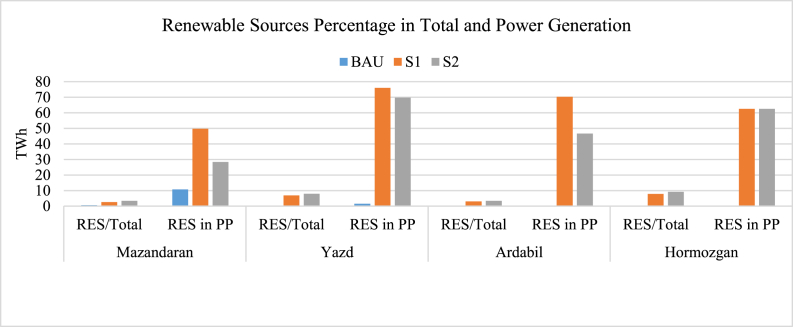
Contribution of renewable resources in PES and power production.

Fig. 10 indicates the share of renewable energy sources (RES) in PES and power production. As indicated, S1 and S2 increase the share of renewable sources greatly. S2 has a higher percentage in RES/PES than S1 since CHP plants produce heating, which leads to lower PES used for heating. Because CHPs should work with a minimum capacity to support the district heating system, S2 has a lower percentage of RES/PP. While in S1, more renewable resources are used, and the heating is supported by non-renewable fuel-burning boilers. For all provinces, RES/PP is considerably higher in S1 and S2 than BAU with a maximum of 40%, 73%, 68%, and 60% difference for Mazandaran, Yazd, Ardabil, and Hormozgan respectively. This shows the great results of the correct policies which could benefit the 2032 energy system.

**Fig. 11:**
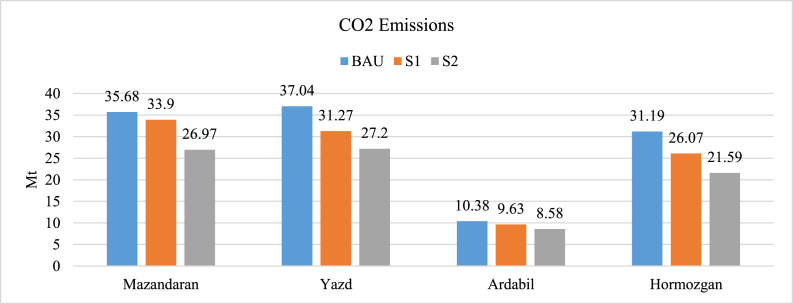
CO_2_ emissions of different scenarios.

Fig. 11 illustrates the CO_2_ emissions of different scenarios in 2032. As shown, S2 has the lowest GHG emissions. So, it is the cleanest scenario. The reduction of BAU to S1 is due to using renewable energy sources, while the reduction of S1 to S2 is due to utilizing district heating instead of boilers. Ardabil has the lowest reduction of 1.8 Mt (17%), while Yazd has the most reduction of 9.84 Mt (27%). Hormozgan has a decrease of 9.6 Mt (31%), and Mazandaran has a decrease of 8.71 (24%). This proves that the policies made are completely environmentally friendly. The influence of weather conditions is such that the potential of renewable energy is greater mainly in hot climates. Also, improving the quality of the fuels could be vastly helpful with CO_2_ emissions.

**Fig. 12:**
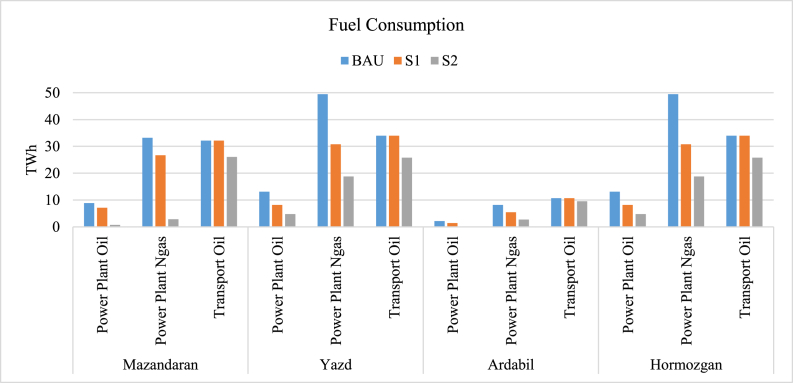
Fuel consumption.

Fig. 12 shows the fuel consumption of power plants and transport systems of all provinces. As shown, in S1 and S2, fuel consumption decreases greatly due to renewable energy power generation and improved fuel quality. Yazd and Hormozgan consume the maximum amount of fuels in power generation in BAU while having the most potential renewable sources. Utilizing S1 and S2 could be vastly beneficial in terms of fuel consumption and thus help sustainability and avoid resource depletion.

### 3.3 Energy policy impacts

The current energy policies in Iran are to level the demand and supply sections. In the demand section, the main objective is to save as much energy as possible by designing periodic power outages in the country during peak hours. Also, regarding the low price of natural gas in the supply section, adding gas-powered power plants is the only selected policy. As discussed before, different scenarios lead to different policies. The potential of renewable energy assessed, changing the type of the old power plants, improving fuel quality, using heat pumps when needed, and utilizing district heating are the policies inserted into the energy system of each province in different scenarios.

**Fig. 13:**
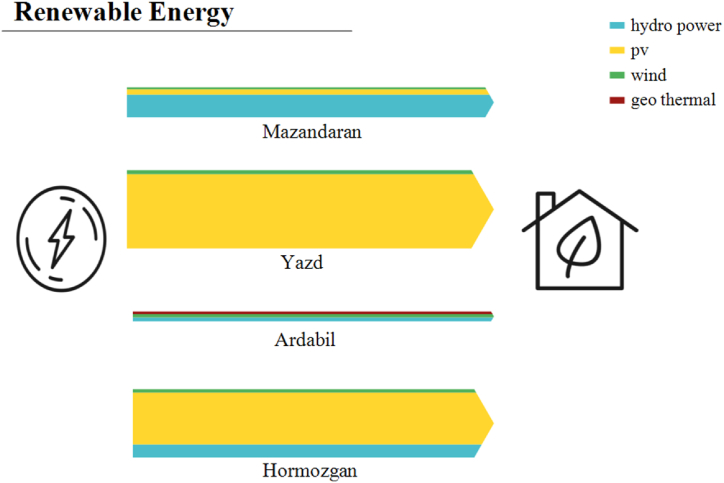
Renewable energy generation in S2.

Fig. 13 illustrates the renewable energy power generation in S2 for the provinces. As mentioned, hot climates benefit from solar energy more than other climates. Hormozgan and Mazandaran use hydropower because of the numerous rivers and dams in the region and their proximity to the seas. Ardabil uses wind and geothermal energy sources due to the wind and geothermal potential. This proves that all forms of renewable energy sources could be utilized in different parts of Iran. Aside from renewable energy, developing the current energy system into the mentioned policies could be energy effective, environmentally friendly, and easy to adapt. Being able to use heat pumps in winter can turn both renewable and non-renewable excess electricity production into the amount of heating needed. Also, being able to use district heating with CHPs could lead to the adaptability of changing the power-heating generation based on their demands. So, correct policies could be chosen for different climates, and if all provinces’ energy systems are correctly improved by 2032, at least 20% of emissions could be avoided while energy shortage would not exist.

## 4 Conclusion

In this study, four provinces of Iran, each located in a different climate zone, were chosen. The past data on energy systems, including heating, cooling, electricity, industry, transport, and power generation, in demand and supply sections were gathered and analyzed. Then the future data is predicted, and the energy system of each province is discussed in 3 different future scenarios of BAU, S1, and S2. The results show the below conclusion. The following are the conclusions based on the results from the current study.

• The performance of solar systems is higher in hot & humid and hot & dry climates, while in cold climates, wind and geothermal resources have higher efficiencies. Also, in temperate & humid climates, hydropower plants are more beneficial. So, policies can vary the renewable supply system based on the climate region.
• The total heating demand of all provinces could be supplied by the waste heat produced in CHP plants, while both renewable and non-renewable power plants provide electricity. This means there would be no energy shortage in any sector in any climate throughout the year.
• Using S1 and S2, the consumption of primary energy sources reduces by at most 50 TWh/year while the share of renewable energy sources in power generation could be increased up to 70 TWh/year. This could positively impact GHG emissions, global warming, and air pollution.
• Using S1 and S2, CO_2_ emissions could be avoided between 17 and 31% in different provinces while no energy shortage happens. This could minimize the temperature rise in Iran caused by global warming.
• The fuel consumption of power plants and transport sectors can be reduced by almost half by choosing S2. This could lead to a more efficient energy system. Also, the remaining fossil fuels could be exported for financial benefits.
• Although all climates benefit from both S1 and S2 scenarios, the hot climates' energy systems could be vastly improved because of the added renewable resources and the optimized future demands. This means that in such climates, changing the energy policy is more important and could have more efficient results.
• Using S1 and S2, Primary energy sources share could increase from 1% to 8% in 2032, considering the additional renewable sources.
• Future studies could discuss many little areas by using more precise location techniques and localizing the energy system for each region based on the resource assessments done.
• Locating the exact location of each renewable power plant could result in better precision of each renewable source.
• Other techniques of data prediction could be implemented to calculate future data more precisely.

## Declarations

### Author contribution statement

Saeed Ahmadi-Kaliji: Conceived and designed the experiments, Wrote the paper

Ahmad Hajinezhad: Contributed reagents, materials, analysis tools or data, Analyzed and interpreted the data

Alireza Kashani Lotfabadi: Conceived and designed the experiments; Wrote the paper; Analyzed and interpreted the data

Reza Fattahi: Analyzed and interpreted the data, Performed the experiments

Seyed Farhan Moosavian: Contributed reagents, materials, analysis tools or data, Wrote the paper

## Data availability statement

Data will be made available on request.

No additional information is available for this paper.

## Declaration of competing interest

The authors declare that they have no known competing financial interests or personal relationships that could have appeared to influence the work reported in this paper.
